# Surgical outcomes of segmental diaphyseal forearm fractures in adults: a small case series on plate osteosynthesis, intramedullary nailing, and other surgical methods

**DOI:** 10.1186/s12891-023-06857-1

**Published:** 2023-09-14

**Authors:** Dong Hee Kim, Hyo Seok Jang, Sang Ho Kwak, Sung Yoon Jung, Jong Min Jeon, Tae Young Ahn, Sang Hyun Lee

**Affiliations:** 1 Departments of Orthopedic Surgery, Samsung Changwon Hospital, Sungkyunkwan University School of Medicine, Changwon, Republic of Korea; 2 Department of Orthopedic Surgery, Inje University Haeundae Paik Hospital, Inje University College of Medicine, Busan, Republic of Korea; 3 Department of Orthopedic Surgery, SNU Seoul Hospital, Seoul, Republic of Korea; 4 Department of Orthopedic Surgery, College of Medicine, Dong-A University Hospital, Busan, Republic of Korea; 5 Department of Orthopedic Surgery, Medical Research Institute, Pusan National University Hospital, Pusan National University School of Medicine, Busan, Republic of Korea

**Keywords:** Segmental, Diaphyseal, Forearm, Fracture

## Abstract

**Background:**

Segmental fractures often result from high-energy or indirect trauma that causes bending or torsional forces with axial loading. We evaluated surgical outcomes of patients with forearm segmental diaphyseal fractures.

**Methods:**

We retrospectively analyzed data from patients with forearm segmental fractures for which they underwent surgery at the Pusan National University Trauma Center from March 2013 to March 2022. We also analyzed accompanying injuries, injury severity score (ISS), injury mechanism, occurrence of open fracture, surgical technique, and treatment results.

**Results:**

Fifteen patients were identified, one with bilateral segmental diaphyseal forearm bone fracture, for a total of 16 cases. Nine of the patients were male. The overall mean age was 50 years, and the mean follow-up period was 16.2 months. Six cases who underwent surgery using plate osteosynthesis achieved bone union without length deformity at final follow-up. Three of seven patients who underwent intramedullary nailing alone underwent reoperation due to nonunion. Six cases achieved bone union at final follow-up, three of which showed length deformity. Three patients underwent surgery using a hybrid method of IM nailing, plates, and mini cables. One patient who underwent surgery with a plate and one patient who underwent surgery with IM nailing alone showed nonunion and were lost to follow-up.

**Conclusion:**

Plate osteosynthesis is considered the gold standard for treatment of adult forearm diaphyseal segmental fractures. In this study, IM nailing was associated with high rates of non-union and length deformity. However, the combination of IM nailing and a plate-cable system may be an acceptable alternative in segmental diaphyseal forearm fracture, achieving a union rate similar to that provided by plate fixation.

## Introduction

The goal of treatment for forearm fracture is to ensure maintenance of optimal length and radioulnar joint relationships with full pronosupination [1–3]. There are various options for treating such fractures, including closed management and surgical interventions. Decisions regarding treatment are based on factors such as fracture pattern, patient age, and soft-tissue envelope integrity [3, 4].

Traditionally, open reduction and internal fixation (ORIF) is the preferred treatment for forearm shaft fracture because it provides adequate fixation force to the fractured area [2–5]. Intramedullary (IM) nails are occasionally used in pediatric patients but are generally not appropriate for adults due to their inability to provide sufficient rotational and linear stability to this region, leading to high non-union rates and need for additional long-term fixation [1]. Early published reports reported non-union rates for surgery using K-wires, Steinman pins, or Ender nails [6]. Following numerous improvements, an interlocking IM nail was developed and currently exhibits adequate surgical outcomes [3, 7–9].

Segmental diaphyseal forearm fracture is an infrequent occurrence, often resulting from high-energy trauma and frequently causing soft tissue injury. In addition, depending on the location of the fracture, the middle fragment may be long and other fragments too short for rigid fixation or double plating. In such cases, an interlocking IM nail can be useful; IM pinning is non-invasive, minimizes soft tissue damage, and is not limited by fragment length [3]. Although adequate results have consistently been reported in the treatment of diaphyseal forearm fractures, the optimal treatment modality has not been established, [4, 7, 8, 10] and few reports describe the outcomes of surgery for segmental fracture in the forearm shaft [7].

In this study, we utilized various surgical interventions to treat segmental fractures in the forearm shaft and report short-term outcomes and complications of surgical treatment of these segmental fractures. In additionally, we introduce a hybrid nail, plate, and cable technique for repairing segmental diaphyseal forearm fracture.

## Materials and methods

This study was approved by the Institutional Review Board (IRB) of the Medical Research Institute of Pusan National University. All procedures were performed in accordance with the relevant guidelines and regulations. A retrospective study of patients diagnosed with forearm segmental fractures who underwent surgery from March 2013 to March 2022 at Pusan National University Severe Trauma Center was conducted. The inclusion criterion was patients with AO classification C1.1 1.2 1.3, C 2.1 2.2.2.3, or C 3.13.2.3.3. Pediatric patients younger than 18 years were excluded. We assessed patient gender and age, accompanying injuries, injury severity score (ISS), injury mechanism, occurrence of open fracture, surgical technique, and treatment results.

Surgeries were performed by two surgeons under general or brachial plexus block anesthesia. Open reduction and plate fixation were performed using the standard surgical method. Radial fractures were mainly treated through the Henry or Thompson approach, and ulnar fractures were treated using a posterior approach. Plates with five or more holes, such as recon-plates or limited contact dynamic compression plates, were used. In cases involving bone marrow fixation, Ender nails (DuPuy Synthes, Raynham, MA, USA) were only used in one case; all other patients were treated with interlocking IM nails (Acumed, Hillsboro, OR, USA). Surgeries were performed using the standard technique according to the manufacturer’s suggestions. The nail used to fix the radius was inserted into the medullary canal through an entry hole created with an awl in the distal end of the radius. The nail used to fix the ulna was inserted into the medullary canal the through a longitudinal 1-cm incision at the tip of the olecranon. Next, a handheld reamer was inserted to ream the canal and the length was measured after the reamer was withdrawn. Nail position was assessed fluoroscopically in orthogonal planes to ensure that the nail successfully crossed the fracture site and maintained good reduction. The nail was then interlocked with a fully threaded 3.5-mm selftapping screw. The plate and cable combination technique were mainly performed at the distal fracture site. An approximately 6 cm incision was made for the radius using the Henry or Thompson approach and for the ulna using the posterior approach. To minimize periosteal detachment, the fracture site and the area where the cable should be placed were dissected. After reduction with a reduction clamp, a 4-hole plate was placed on the fracture site and both sides were fixed with cables. When applied to the nonunion area that occurred after IM nailing alone, the gap was temporarily fixed with screw fixation and then reduced using a screw holder or bone graft before fixing the plate.

In the three patients in whom the nail-plate-cable hybrid technique was used, the IM nail fixation, four-hole small recon plate, and 1.0 mm mini-cable (DuPuy Synthes) method were used.

Definitive surgery was delayed up to one month depending on the patient’s overall condition. Cases that presented as emergencies were not able to undergo the complete surgical procedure. In such cases, we recorded the surgical method as the last bone fixation method used. A long-arm splint or cast was maintained for six weeks after surgery. At six weeks postoperatively, the elbow brace was removed and active forearm supination and pronation exercises were allowed.

All patients included in this study were followed for at least six months. Fracture union was defined as absence of signs of non-union on anteroposterior and lateral radiographic views. Before and after surgery, the length of the ulna was compared with that of the contralateral ulna using radiographic images. Functional outcomes were investigated using medical records regarding pain in the forearm and wrist, as well as the ability to return to daily life and work.

## Results

Fifteen patients with segmental forearm fractures who underwent surgery at Pusan National University Trauma Center from March 2013 to March 2022 were identified; nine were male and six were female. The mean age was 50 years (range, 20–74 years), and the mean follow-up period was 16.2 months (range, 6.2–22.7 months). The average ISS was 21.9 points (range, 4–48 points), and nine patients had experienced severe trauma with an ISS of 15 or higher. Among the cases involving accidents, eight were traffic accidents (four of which were pedestrian accidents), three were falls, two were crushing injuries caused by machinery, one was a slip, and one was an explosion. Eleven fractures occurred on the left forearm. Among the 15 patients, one had bilateral segmental diaphyseal bone fracture. Among the admitted patients, seven had open fractures: five Gustilo-Anderson type I, one type II, and one type III (Table 1).

**Table 1:** Demographics and surgical characteristics of patients with diaphyseal segmental forearm fracture

Sex	Age	Mechanism	ISS	Combined Orthopedic Fractures	Other part injury	Open Fx. (G-A type)		Forearm Fracture		First Operation Method	Second Operation Methods		Final outcome (Radiological)		Functional outdome	Follow-up periods (months)	
							Side	Radius (R)	Ulnar(U)								
M	28	Explosion	17	Fx. 3rd MC base Lt. F. 4th prox. phalanx base Lt.	BOF Frontal sinus fx both F. Depressed skull fx Rt T		Lt	Intact	Segmental	Endernail				Union	Pain, return to work	19.5	
F	68	Incar TA	43	Lt. upper arm laceration Fx. neck femur and distal femur Lt. open Fx. patellar Both Fx. proximal tibia Both	Basal skull Fx. Pneumocephalus Lt. fronral skull Fx SDH, SAH C3 body Fx.	II	Lt	Shaft	Segmental	Interlocking IMN				Union: Ulnar positive	Wrist pain, good activities of daily living	8.0	
M	64	Pedestrian TA	48	Lt. hand degloving injury Multiple finger Fx. Fx. neck femur shaft Lt. Pelvic ring injury Fx. sacral Rt.	SDH MRF Diaphragm injury, Liver laceration, Pancreas contussion	I	Lt	Segmental	Segmental	Dual plate(radius), Single plate(ulnar)				Union	Pain, finger contracture, Unable to return to work due to pelvis and leg pain	22.7	
M	58	Fall down	14	Pelvic ring injury Fx. acetabular Rt.	L2 transverse process, Rt.		Rt	Intact	Segmental	Interlocking IMN and plate-cable combinateion				Union	No pain, return to work	17.7	
F	39	Fall down	17	Fx. proximal clavicle Lt. Pelvic ring Injury Fx. distal femer Lt. Lt. foot 5th MT fx.		I	Lt	Fx. distal radius and Fx. radial neck	Segmental (3 site fracture)	Interlocking IMN		Plate- cable cobimination + Iliac bone graft		Union:Ulna positive	Pain, Finger contracture, unable to return to work due to pelvis pain	18.2	
M	42	Incar TA	27		MRF, Lung contusion Pan facial bone Fx.	I	Lt	Segmental	Shaft	Interlocking IMN and plate-cable combinateion		Plate- cable cobimination + Iliac bone graft		Union	No pain, return to work	13.7	
F	53	Fall down	24	Fx. bodyscapula Rt. Fx. supracondylar humerus Rt. Brahical artery injury	MRF Multiple sinous process fx.		Rt	Shaft	Segmental	Interlocking IMN and plate-cable combinateion				Union	Impossible to evaluate due to schizophrenia and radial nerve palsy	12.0	
M	74	Pedestrian TA	-	-	-		Lt	Shaft	Segmental	Dual plate				Nonunion: Follow-up Loss		6.2	
M	20	Machinary injury	16	open Fx. shaft humerus Lt.	-	III	Lt	Shaft	Segmental	Interlocking IMN				Nonunion: Follow-up Loss		6.2	
M	53	Motorcycle TA	36	Pelvic ring injury Fx. acetabular Rt.	Spinal cord contusion C3/4		Rt	3 Fragment	styloid	Interlocking IMN		IM nail remove, ORIF /c plate, Iliac bone graft		Union: Radius shortening	Impossible to evaluate due to quedriphresia	32.2	
M	29	Pedestrian TA	22	Pelvic ring injury, Fx. acetabular Lt.	Mandible Fx. Maxillar Fx. Sternum Fx. Cerebral contusion		Lt	3 Fragment	Intact	Interlocking IMN		IM nail remove, ORIF /c plate, Ulnar shortening osteotomy		Union	limited rotation of forearm, return to work.	27.6	
M	42	Pedestrian TA	14	-	Cerebral contusion Nasal bone Fx. Rt. BOF Maxillary Fx.	I	Rt	Shaft	3 Fragment	Single plate				Union	No pain, return to work	11.0	
F	54	Machinary injury	4	Fx. shaft humerus Lt.	-		Lt	shaft and distal	3 Fragment	Single plate				Union	Pain, return to work	17.1	
F	67	Incar TA	12	Fx. supracondylar humerus Lt. Fx. acetabular post. wall Rt.	-		Lt	Shaft	3 Fragment	Dual plate				Union	Pain, return to work	21.8	
F	58	Slip down	12	-	-	I	Lt	Shaft	3 Fragment	Interlocking IMN				Union	No pain, good activities of daily living	20.0	

GA: Gustilo-Anderson TA: traffic accident, Rt: Right, Lt: Left, Fx.: Fracture, MT: Metatarsla bone, C: cervical spine, L: Lumbar spine BOF: Blow Out Fractures, SDH: subdursal Hemorrhage, SAH: Subacromial Hemorrhage,MRF: Multiple Rib Fractures, IMN: Intramedullary nailing, ORIF: Open reduction and internal fixation

There were no cases of postoperative infection, compartment syndrome, or neurovascular injury due to surgery. One case required split thickness skin graft due to soft tissue damage caused by the initial trauma.

Open reduction and plate fixation were performed in six patients; three required the use of a single plate, while three required dual plates. Seven cases required bone fixation using IM nailing; one underwent closed reduction and fixation with an Ender nail, and six cases were performed using interlocking IM nails, three of which required interlocking IM nailing and plate-cable placement.

**Fig. 1:**
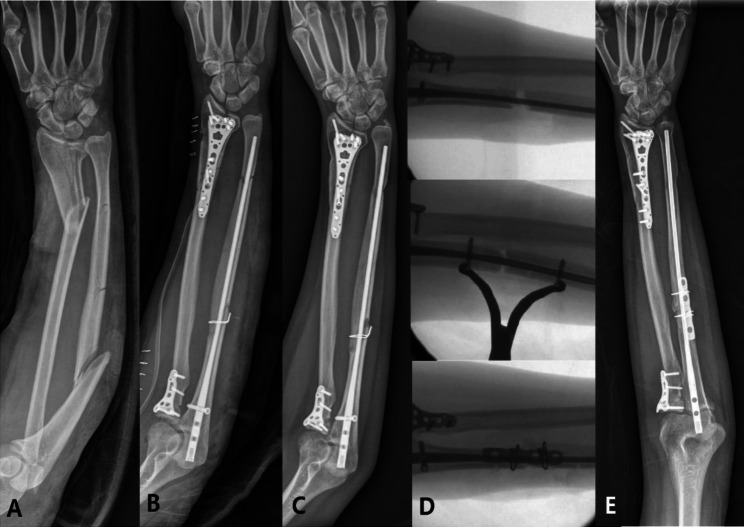
Serial photographs of a 39-year-old female patient showing segmental fracture of the ligament ulna and fractures of the distal radius and radial neck due to a fall. Interlocking intramedullary (IM) nailing was performed for an ulnar fracture; however, proximal non-union was observed at seven months after surgery. Bone union was achieved at 13 months after additional fixation surgery using a plate and cable

**Fig. 2:**
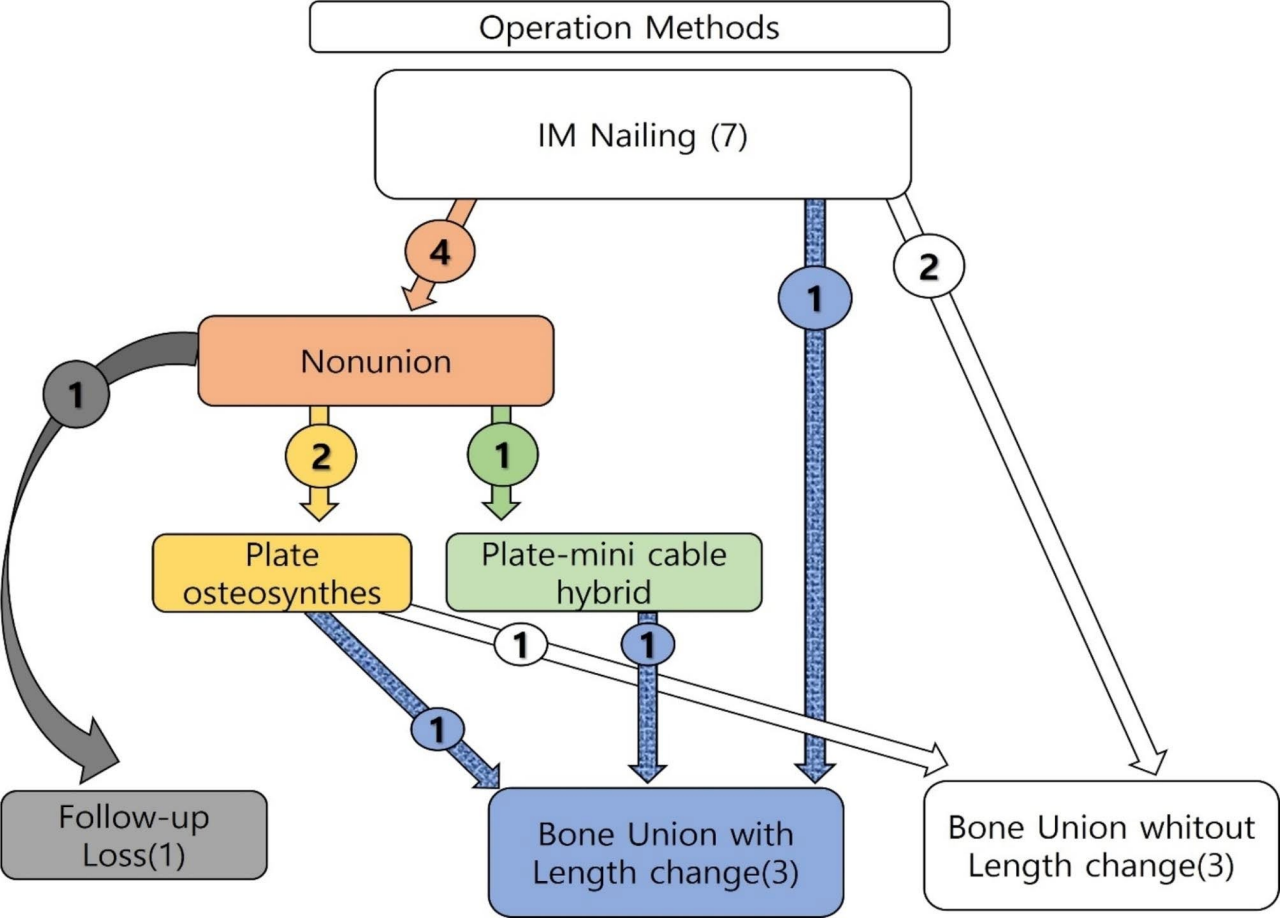
Outcomes of cases that underwent surgery using only intramedullary (IM) nailing

During follow-up, three of seven patients who only underwent IM nailing showed signs of non-union and underwent reoperation. Two cases that underwent surgery using a hybrid method of IM nailing, plating, and mini-cable placement with auto-bone graft (Fig. 1) and one case that underwent ORIF with one plate and auto-bone graft achieved bone union at final follow-up. One patient was transferred to another hospital (Fig. 2).

**Fig. 3:**
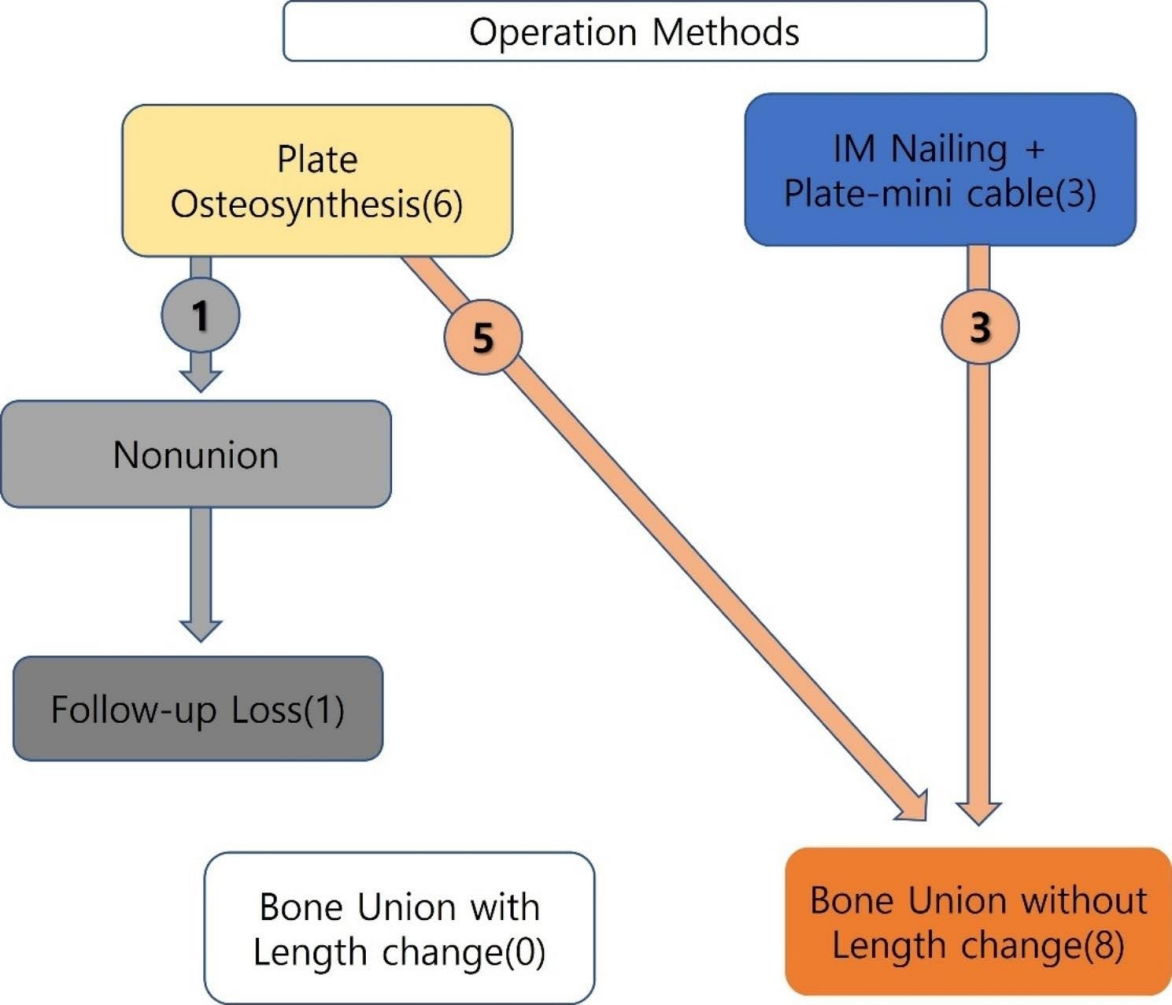
Outcomes of cases that underwent surgery using plate osteosynthesis and intramedullary (IM) nailing-plate-cable combination

Three cases that underwent surgery using a hybrid method of IM nailing, plates, and mini-cables, as well as three cases that underwent ORIF with one plate, all achieved bone union. Six cases (five patients) who underwent surgery using plate osteosynthesis achieved bone union. One patient who underwent surgery using dual plates showed nonunion at postoperative six months and transferred to another hospital (Fig. 3). Among the five cases of non-union, two were open fractures.

**Fig. 4:**
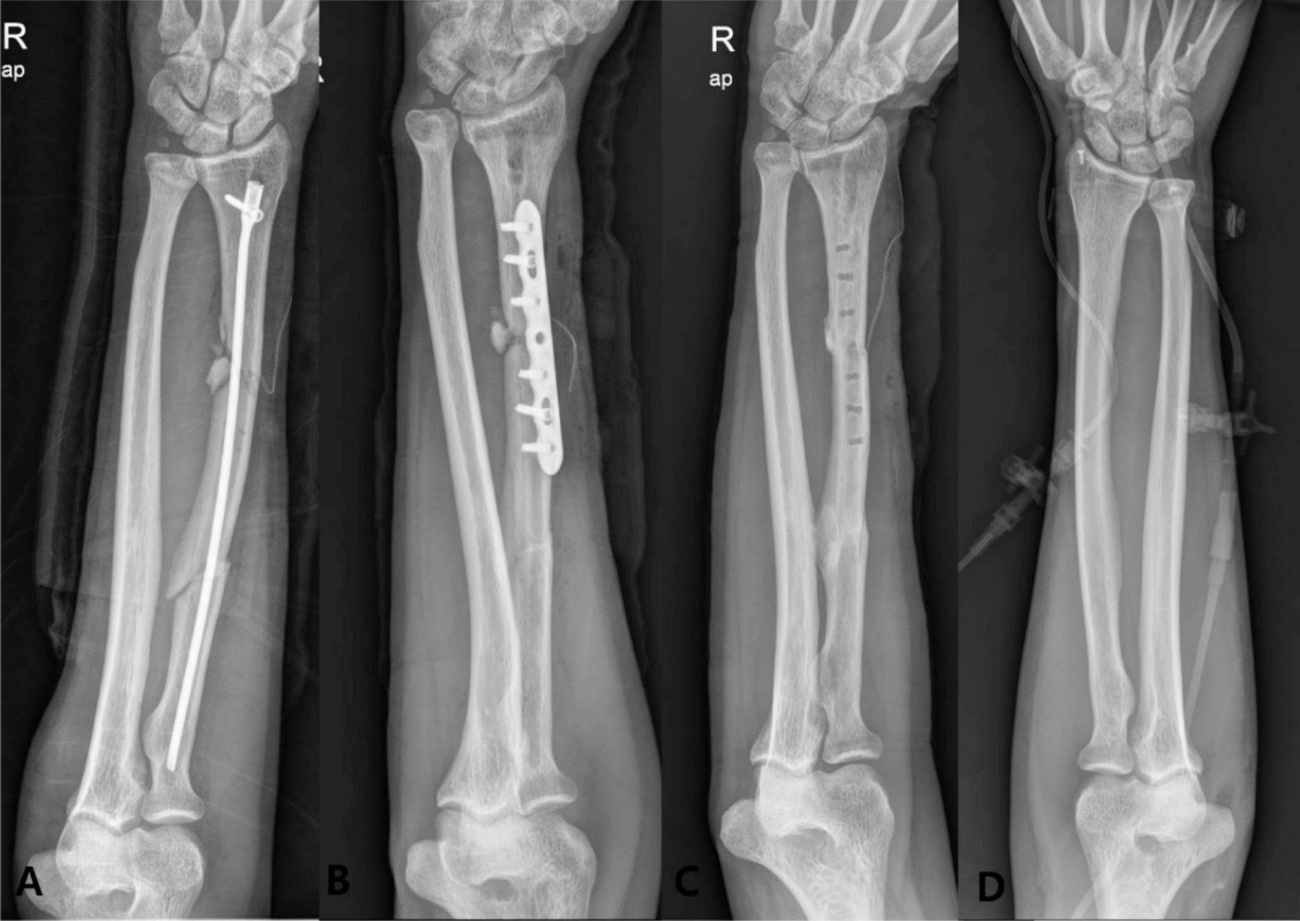
(**A**) Photograph of a 53-year-old male patient who had a segmental fracture of the right radial shaft due to a traffic accident. Surgical treatment was performed using interlocking intramedullary (IM) nailing. (**B**) Open reduction, internal fixation, and autogenous bone graft were performed on the non-union in the distal part of the radius at six months after surgery, and (**C**) bone union was achieved. Shortening of the radial length was observed compared with the unaffected side (**D**)

Among the 14 patients who achieved bone union by the final visit, all three cases in which the plate osteosynthesis and interlocking IM nails-plate-cable hybrid method was used showed no length changes and achieved primary bone union. Three cases in which only interlocking IM nailing was used at the first surgery showed length changes at final follow-up (Fig. 4).

When patients reported pain and irritation due to implants, or felt uncomfortable with metal in their body, implant removal was performed upon request. A total of five patients underwent implant removal surgery after obtaining union.

## Discussion

In this study, ORIF with plates showed the best results for bone union, and interlocking IM nailing alone resulted in a higher incidence of non-union. Even though only 3 patients were treated using the IM nail and plate-cable hybrid technique, it also showed good results. Although we included patients with relatively high-energy injuries, there were no complications such as surgical infection, soft tissue necrosis, or compartment syndrome. ORIF affects anatomical reduction and produces the best union rate and future function. Bridge plating methods have also been used in more complex fracture patterns, [3, 5, 11] and compression plate techniques are the surgical methods of choice in simple adult forearm fracture patterns [2]. In cases with extensive open wounds or when the length of the middle bone segment in segmental fractures is too long to position a plate to sufficiently stabilize the fracture, dual plates may be used. However, dual plating may cause mechanical failure due to stress shielding and can be resolved with IM nailing [7].

IM nailing is a standard treatment for fractures of the long bone shaft and mainly is used for cases involving polytraumatic high-energy long-bone fractures, osteoporotic bone fractures, or pathological fractures [12, 13]. IM nail techniques minimize damage to soft tissue and the periosteum and promote secondary callus formation, providing a better alternative. The recently developed interlocking IM nailing method prevents not only rotational stability of the fracture, but also bone shortening [8, 9, 14, 15]. Some researchers have suggested that comminuted fractures, segmental fractures, and fractures near the diaphyseal-metaphyseal junction are appropriate indications for interlocking IM nailing in forearm fractures [3, 5, 7, 16].

However, in our study, IM nailing alone resulted in a higher incidence of non-union and changes in bone length. Polat et al. [14] compared two groups who were treated with IM nails and plates, finding that distant locked IMNs are a viable alternative to ORIF with plate osteosynthesis with similar healing rates, functional scores, and shorter operative times. In that study, there were only three patients with diaphyseal fractures, making it difficult to conclude that this method is also useful for treating segmented fractures. In other previous studies, IM nailing in forearm bone fractures was used for simple, non-segmental fractures [4, 9, 10, 14, 15, 17]. Our results were different from previous results, and are the core of our study implications. It is difficult to directly compare the results of the present and previous studies. Differences in intramedullary nail products used in different studies may explain these discrepancies. The design of the intramedullary nail curve may differ from product to product, which could in turn result in differences in fixation strength. However, a major difference is that in our study we included only targeted patients with diaphyseal segmented fractures. Diaphyseal segmented fractures of the forearm are caused by high-energy damage, which can have a negative impact on bone healing. In addition, most of our patients presented with segmental shaft fractures and distal or proximal fractures of the other bones, rather than single segmental shaft fractures, and were therefore less likely to have lower forearm stability. This may be because IM correction alone is not stable enough for these types of fractures. Based on our experience, we hypothesized that IM nailing will not prevent rotational and linear stability in segmental fractures [17]. In the process of treating forearm segmental shaft fractures with IM nailing, reoperation is frequently performed due to the lack of stability of the fracture. Therefore, we chose a method to increase stability by adding a small plate and mini-cable to interlocking IM nailing.

The combination nail and plate technique has been shown to be useful in femoral or tibial fractures [18, 19]. [20] However, wide use of this technique in the upper extremities is impractical because bone diameters are so small that screw fixation is difficult to avoid. We performed the IM nailing technique and the plate-cable combination technique instead of plate-screw fixation. Our preoperative assessments indicated that there was not enough space to insert screws without using intramedullary nails. Therefore, we fixed the distal part of the segmental fracture with a plate-cable to form a fragment pattern that promoted bone union. We hypothesized that applying additional plate-cable systems to unstable fracture sites would be more effective than IM nails alone. However, when the fracture site is located in proximal rather than distal areas of the forearm, surgery becomes more difficult and time-consuming, and soft tissue damage can be increased. Therefore, we performed additional procedures in distal fracture sites only to maintain the advantages of using IM nails, and fortunately obtained good results. We found that providing sufficient stability to obtain bone union can be achieved simply by fixing any additional area. In addition, bone union without changes in bone length was obtained in primary operations using interlocking nailing and plate-cable hybrid techniques, indicating sufficient linear stability with the plate-cable combination method. We suggest that this hybrid technique is mainly useful for segmental fractures of the shaft of the forearm caused by high-energy injury, especially when segmental fractures of the ulna or radius are accompanied by fractures of other bone on the same side (e.g., segmental fractures of the shaft of the ulna with proximal radial fractures or segmental fractures of the radius with olecranon fractures of the ulna).

Although valuable, the present study has limitations that should be acknowledged. First, the patient sample was relatively small, causing difficulty in drawing significant conclusions regarding surgical results. In particular, there were only 3 patients who were treated by the hybrid technique, and it was therefore difficult to identify the advantages and disadvantages of this technique. In addition, prognosis could not be classified according to whether the fractured bone was radial or ulnar. However, even if most patients have segment fractures on one site, there are many cases in which another bone also sustains a distal or proximal fracture, so it is difficult to make realistic comparison according to whether the radius or ulna are involved. Third, the range of motion and upper limb function scores were not measured, which may limit the overall scope of the study. Furthermore, while range of motion in the forearm is an important measure of surgical success, the most severe cases in this study had limited motion due to their injuries or other underlying medical conditions. Consequently, obtaining accurate range of motion or score data was challenging. Detailed investigations such as operation time, time to bone union, patient satisfaction, and range of motion are required, and additional research is needed to collect and compare more cases according to each surgical method. Despite these limitations, this study provides valuable insights into the surgical outcomes of patients with similar conditions and serves as a foundation for future research in this area.

## Conclusions

This study supports plate osteosynthesis as the best treatment method for diaphyseal segmental forearm fractures in adults. The IM nailing technique alone showed a high rate of non-union and length deformity. The interlocking nailing and plate and cable hybrid technique resulted in a union rate similar to that provided by plate fixation. Therefore, the choice of using only IM nailing for segmental forearm fractures should be made with caution, and the combination using a plate-cable fixation can be an acceptable assistive surgical method in segmental diaphyseal forearm fracture.

## Data Availability

The datasets used and analyzed during the current study are not publicly available due to lack of participant consent to share their data but are available from the corresponding author upon reasonable request after ethical considerations are met.

## References

[1] McAuliffe JA (1997). Forearm fixation. Hand Clin.

[2] Bartonicek J, Kozanek M, Jupiter JB (2014). History of operative treatment of forearm diaphyseal fractures. J Hand Surg Am.

[3] Rehman S, Sokunbi G (2010). Intramedullary fixation of forearm fractures. Hand Clin.

[4] Kose A, Aydin A, Ezirmik N, Yildirim OS (2017). A comparison of the treatment results of dpen reduction internal fixation and intramedullary nailing in adult forearm diaphyseal fractures. Ulus Travma Acil Cerrahi Derg.

[5] Moss JP, Bynum DK (2007). Diaphyseal fractures of the radius and ulna in adults. Hand Clin.

[6] Sage FP, Smith H (1957). Medullary fixation of forearm fractures. J Bone Joint Surg Am.

[7] Gao H, Luo CF, Zhang CQ, Shi HP, Fan CY, Zen BF (2005). Internal fixation of diaphyseal fractures of the forearm by interlocking intramedullary nail: short-term results in eighteen patients. J Orthop Trauma.

[8] Lee YH, Lee SK, Chung MS, Baek GH, Gong HS, Kim KH (2008). Interlocking contoured intramedullary nail fixation for selected diaphyseal fractures of the forearm in adults. J Bone Joint Surg Am.

[9] Saka G, Saglam N, Kurtulmus T, Avci CC, Akpinar F, Kovaci H (2014). New interlocking intramedullary radius and ulna nails for treating forearm diaphyseal fractures in adults: a retrospective study. Injury.

[10] Kale SY, Singh SD, Samant P, Bukalsaria D, Chaudhari P, Ghodke RJ (2021). Treatment of diaphyseal forearm fracture with interlocking intramedullary nailing: a pilot study. J Clin Orthop Trauma.

[11] Schulte LM, Meals CG, Neviaser RJ (2014). Management of adult diaphyseal both-bone forearm fractures. J Am Acad Orthop Surg.

[12] Baltov A, Mihail R, Dian E (2014). Complications after interlocking intramedullary nailing of humeral shaft fractures. Injury.

[13] Putti AB, Uppin RB, Putti BB (2009). Locked intramedullary nailing versus dynamic compression plating for humeral shaft fractures. J Orthop Surg (Hong Kong).

[14] Polat O, Toy S (2022). Comparison of the clinical and radiographic outcomes of plate fixation versus new-generation locked intramedullary nail in the management of adult forearm diaphyseal fractures. Acta Orthop Traumatol Turc.

[15] Lee SK, Kim KJ, Lee JW, Choy WS (2014). Plate osteosynthesis versus intramedullary nailing for both forearm bones fractures. Eur J Orthop Surg Traumatol.

[16] De Pedro JA, Garcia-Navarrete F, Garcia De Lucas F, Otero R, Oteo A. Lopez-Duran Stern L. Internal fixation of ulnar fractures by locking nail. Clin Orthop Relat Res. 1992(283):81–5. 1395274

[17] Al-Sadek TA, Niklev D, Al-Sadek A (2016). Diaphyseal Fractures of the forearm in adults, plating or Intramedullary Nailing is a better option for the treatment?. Open Access Maced J Med Sci.

[18] Liporace FA, Yoon RS (2019). Nail plate combination technique for native and periprosthetic distal femur fractures. J Orthop Trauma.

[19] Hussain MS, Dailey SK, Avilucea FR (2018). Stable fixation and Immediate Weight-Bearing after Combined Retrograde Intramedullary Nailing and Open Reduction Internal fixation of noncomminuted distal interprosthetic femur fractures. J Orthop Trauma.

[20] Kanabur P, Sandilands SM, Whitmer KK, Owen TM, Coniglione FM, Shuler TE (2017). Nail and locking plate for Periprosthetic Fractures. J Orthop Trauma.

